# The Glutathione *S*-Transferase *PtGSTF1* Improves Biomass Production and Salt Tolerance through Regulating Xylem Cell Proliferation, Ion Homeostasis and Reactive Oxygen Species Scavenging in Poplar

**DOI:** 10.3390/ijms231911288

**Published:** 2022-09-25

**Authors:** Hongsheng Gao, Chunyan Yu, Ruichao Liu, Xiaoyan Li, Huiqing Huang, Xueting Wang, Chao Zhang, Ning Jiang, Xiaofang Li, Shuang Cheng, Hongxia Zhang, Bei Li

**Affiliations:** 1 The Engineering Research Institute of Agriculture and Forestry, Ludong University, 186 Hongqizhong Road, Yantai 264025, China; 2 Shenzhen Branch, Guangdong Laboratory of Lingnan Modern Agriculture, Genome Analysis Laboratory of the Ministry of Agriculture and Rural Affairs, Agricultural Genomics Institute at Shenzhen, Chinese Academy of Agricultural Sciences, Shenzhen 518120, China

**Keywords:** glutathione *S*-transferase (GST), *PtGSTF1*, reactive oxygen species, salt stress, transcriptomic analysis

## Abstract

Glutathione *S*-transferases (GSTs) play an essential role in plant cell detoxification and secondary metabolism. However, their accurate functions in the growth and response to abiotic stress in woody plants are still largely unknown. In this work, a Phi class Glutathione *S*-transferase encoding gene *PtGSTF1* was isolated from poplar (*P. trichocarpa*), and its biological functions in the regulation of biomass production and salt tolerance were investigated in transgenic poplar. *PtGSTF1* was ubiquitously expressed in various tissues and organs, with a predominant expression in leaves and inducible expression by salt stress. Transgenic poplar overexpressing *PtGSTF1* showed improved shoot growth, wood formation and improved salt tolerance, consistent with the increased xylem cell number and size under normal condition, and the optimized Na^+^ and K^+^ homeostasis and strengthened reactive oxygen species scavenging during salt stress. Further transcriptome analyses demonstrated that the expressions of genes related to hydrolase, cell wall modification, ion homeostasis and ROS scavenging were up- or down-regulated in transgenic plants. Our findings imply that *PtGSTF1* improves both biomass production and salt tolerance through regulating hydrolase activity, cell wall modification, ion homeostasis and ROS scavenging in transgenic poplar, and that it can be considered as a useful gene candidate for the genetic breeding of new tree varieties with improved growth under salt stress conditions.

## 1. Introduction

Glutathione *S*-transferases (GSTs, EC 2.5.1.18) are an ancient super family of catalytic and binding proteins widely distributed in bacteria, plants and animals [1,2]. They are closely associated with biotic and abiotic stresses [3,4,5]. In eukaryotes, based on their cellular locations, GSTs are divided into at least three major protein families, namely cytosolic GSTs, mitochondrial GSTs and microsomal GSTs [6]. Plant GSTs are mainly cytosolic localized and represent up to 2% of soluble proteins [7]. In photosynthetic organisms, based on genomic and phylogenetic analyses, the GST family has been subdivided into 14 classes and the two most widespread types are Tau GSTs and Phi GSTs (GSTFs) [8]. They catalyze the conjugation of a diverse range of xenobiotics and detoxified selective herbicides in the form of dimers [9].

The first plant GSTs were reported in sorghum and maize, and catalyzed the detoxification of the herbicide atrazine by conjugating to the endogenous tripeptide glutathione (GSH, γ-L-glutamyl-L-cysteinyl-glycine) [10]. Since then, a number of studies focused on the detoxification function of glutathione transferase to herbicides in plants have been carried out [11,12,13,14,15,16]. Plant GSTs were also found to be involved in other processes such as plant development and secondary metabolism [17,18]. Expression of *GSTs* is up-regulated under both biotic and abiotic stress conditions, such as high salt, hypoxia, osmotic dehydration, and exposure to safeners and oxylipins [19,20,21,22,23,24,25]. The common effects of these stress conditions are that they all trigger the production of reactive oxygen species (ROS), which leads to oxidative stress to plants. The induction of *GSTs* strongly reinforces their protective role against oxidative stress across this extensive span of treatments [26]. Apart from this, *GSTs* are also induced by phytohormones such as auxin, salicylic acid (SA), abscisic acid (ABA), ethylene, and other hormones, which implies that plant *GSTs* play dynamic roles in plant growth and development [16,27,28]. Increasingly, evidence has demonstrated that unique combinations of multiple signaling pathways from various phytohormones and reactive oxygen species or antioxidants rendered distinct transcriptional activation patterns of individual GSTs during various stresses. Underestimation of post-transcriptional regulation of individual GSTs and the roles of phytohormones (i.e., ABA) in these processes have been discussed [29,30].

To date, a great number of glutathione transferase genes have been found to be involved in plant growth, development and response to biotic and abiotic stresses. In weed black grass (*Alopecurus myosaroides*), the phi class *AmGSTF1*, which possesses GPOX activity, was found to be highly active in herbicide resistance [27]. In Arabidopsis, the phi class *AtGSTF2* contributed the resistance to phenol treatment. Overexpression of *AtGSTF2* increased the tolerance to oxidative stress caused by phenol treatment in transgenic plants [28]. Another tau class GST gene, *AtGSTU17*, was found to be involved in seedling development, hypocotyl elongation, anthocyanin accumulation and far-infrared light-mediated greening inhibition. RT-PCR analyses showed that the expression of *AtGSTU17* was regulated by various photoreceptors, especially phytochrome A (phyA) and different plant hormones, such as auxin and ABA, under all light conditions [31]. Similarly, *GSTU7* was induced by a variety of stress stimuli and played a role not only in the growth of Arabidopsis but also in the protective response for cellular detoxification to herbicides [16]. In Chinese cabbage, the *BcGSTU* gene promoted growth, development and stress tolerance in transgenic Arabidopsis [32]. In rice, *OsGSTU4* encoded a glutathione transferase of tau class. Under salinity and oxidative stress conditions, ectopic expression of *OsGSTU4* in Arabidopsis promoted growth and GST activity, and lowered ROS accumulation in transgenic plants [33]. In addition, ectopic expression of the rice lambda GST gene *OsGSTL2* accelerated growth and development, especially seed germination, of transgenic Arabidopsis plants [34].

In *Populus*, a model woody tree, a total of 81 GST genes have been identified in the annotated genome, including 8 GSTF genes, which contain a two-intron/three-exon structure with similar exons length and a highly conserved first intron position [35]. The expression of *PtGSTF1* has been shown to be induced by various stresses. When poplar plants were exposed to the tent caterpillar *Malacosoma disstria*, or treated with CDNB, H_2_O_2_ or drought, *PtGSTF1* expression was upregulated [35,36,37]. Proteomic studies showed that in poplar plants exposed to cadmium, chilling or salt stresses, GSTF1 protein level was elevated [38,39,40].

A large number of studies on GSTs have been performed in annual herbaceous plants. Knowledge on the specific functions of GSTs in the growth and resistance to abiotic stress in woody plants is still limited, however. In this study, we report the biological role of *PtGSTF1* in the growth and response to high salt stress in poplar. Our study provides new ideas for the breeding of woody plants with both improved growth rate and salt tolerance.

## 2. Results

### 2.1. PtGSTF1 Encodes a Cytoplasmic Phi Class Glutathione S-Transferase with Salt Stress Induced Expression in Poplar

**Figure 1:**
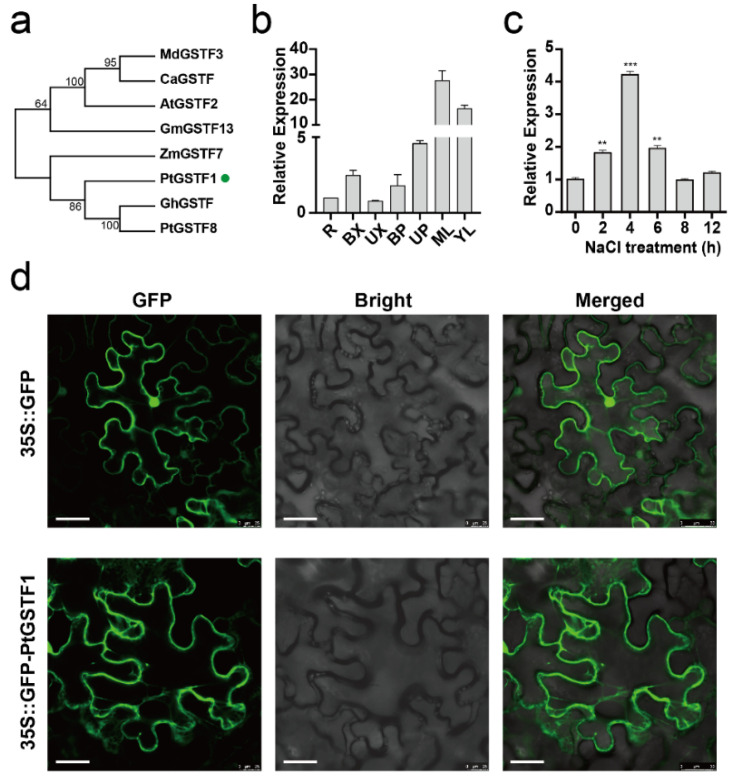
*PtGSTF1* gene expression and protein subcellular localization. (**a**) Phylogenetic tree of *PtGSTF1* (Green dot callout) and GSTF members in different plants. The evolutionary history was inferred using the Minimum Evolution method. The percentages of replicate trees in which the associated taxa clustered together in the bootstrap test (1000 replicates) are displayed next to the branches. (**b**) Quantitative RT-PCR (qRT-PCR) analyses. *PtGSTF1* expression in different tissues and organs of eight-week-old wild type Shanxin yang plants was determined. *PtEF1β* was used as an internal control. R, roots; BX, xylem tissues at the basal position of stems; UX, xylem tissues at the upper position of stems; BP, phloem tissues at the basal position of stems; UP, phloem tissues at the upper position of stems; ML, mature leaves; YL, young leaves. Error bars represent the standard deviation (SD) of three biological replicates. (**c**) *PtGSTF1* expression in response to salt stress. Four-week-old wild type Shanxin yang plants were treated with 150 mM NaCl for 2, 4, 6, 8 and 12 h. (**d**) Subcellular localization of 35S::GFP and 35S::GFP-*PtGSTF1* fusion proteins in tobacco (*Nicotiana benthamiana*) leaf epidermal cells, scale bar = 50 µm. **, *p* < 0.01; ***, *p* < 0.001.

To understand the biological function of GSTs in woody plants, we isolated the coding sequence of *PtGSTF1* from Shanxin yang and compared its amino acid sequence with the GST members from different plant species. Multiple sequence alignment and phylogenetic tree analyses demonstrated that PtGSTF1 shared 53–59% amino acid similarity with its homologous genes from other plants, such as Arabidopsis (*Arabidopsis thaliana*), cotton (*Gossypium spp.*), soybean (*Glycine max*), apple (*Malus domestica*), maize (*Zea mays* L.) and Cathaya (*Cathaya argyrophylla*) (Figure 1a and Figure S1). We then performed qRT-PCR analysis and examined the transcription levels of *PtGSTF1* in different tissues and organs of eight-week-old Shanxin yang plants. We found that *PtGSTF1* was ubiquitously expressed in all the test tissues and organs, including young leaves (YL), mature leaves (ML), upper position xylem (UX), basal position xylem (BX), upper position phloem (UP), basal position phloem (BP) and roots (R), with a predominant expression in young and mature leaves (Figure 1b).

Previous proteomic study has demonstrated an increased *PtGSTF1* protein level in the leaves of *Populus cathayana* male cuttings exposed to salt stress [38]. We investigated the response of *PtGSTF1* in four-week-old Shanxin yang plants treated with 150 mM NaCl for 2, 4, 6, 8 and 12 h. The transcription of *PtGSTF1* was dramatically induced by salt stress. It increased after 2 h and reached the highest level after 4 h (Figure 1c). To see the subcellular localization of PtGSTF1 protein, the *Agrobacterium tumefaciens* strain GV3101 harboring 35S::GFP or 35S::GFP-*PtGSTF1* was introduced into the leaf epidermal cells of *Nicotiana benthamiana*. The GFP fluorescence signal in the leaf cells infiltrated with 35S::GFP was ubiquitously detected including cell membrane and nucleus, whereas GFP fluorescence signal in the leaf cells infiltrated with 35S::GFP-*PtGSTF1* was only observed in cytoplasm, suggesting that PtGSTF1 protein was cytoplasmic localized (Figure 1d).

### 2.2. Transgenic Poplar Plants Overexpressing PtGSTF1 Are Generated

**Figure 2:**
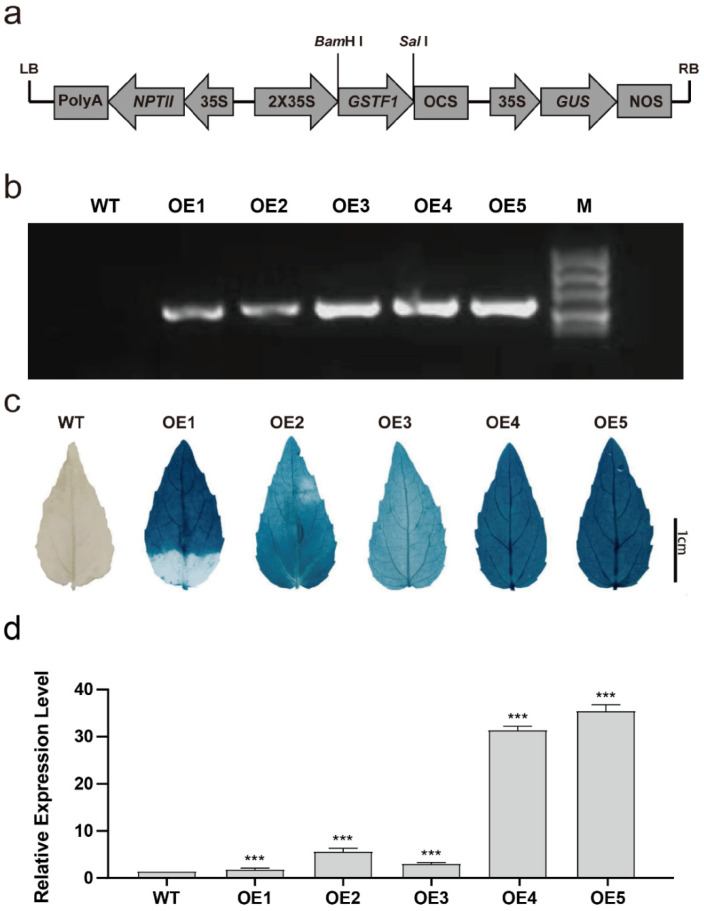
Generation and molecular confirmation of transgenic poplars. (**a**) Diagram of the *PtGSTF1* overexpression vector. Expression of *PtGSTF1* was driven by the cauliflower mosaic virus 35S (*CaMV 35S*) promoter. (**b**,**c**) PCR and GUS staining analysis of wild type (WT) and different independently regenerated *PtGSTF1* transgenic lines. (**d**) qRT-PCR analysis of *PtGSTF1* expression in WT and different transgenic lines. WT, wild type; OE1, OE2, OE3, OE4 and OE5, different transgenic lines overexpressing *PtGSTF1*. Values are the mean ± SD (standard deviation) from three independent experiments (*n* = 3). ***, *p* < 0.001.

To gain an insight into the role of *PtGSTF1* in the growth and stress response of woody plants, the coding sequence of *PtGSTF1* was constructed into the plant expression vector pCAMBIA2301, driven by the cauliflower mosaic virus 35S (*CaMV 35S*) promoter (Figure 2a). The resultant pCAMBIA2301-35S::*PtGSTF1* vector was introduced into the genome of Shanxin yang via Agrobacterium mediated transformation. A total number of 37 independently regenerated kanamycin resistant lines were obtained. The successful integration of *PtGSTF1* into the poplar genome was first confirmed with PCR analyses (Figure 2b). Then, GUS staining and qRT-PCR, respectively, confirmed the expression of GUS and the overexpression of *PtGSTF1* in all the selected transgenic lines (Figure 2c,d). Therefore, three representative transgenic lines with relatively low, medium and high *PtGSTF1* overexpression (OE2, OE4 and OE5) were selected for subsequent growth and stress response analyses.

### 2.3. PtGSTF1 Promotes Shoot Growth and Wood Formation in Transgenic Poplar

**Figure 3:**
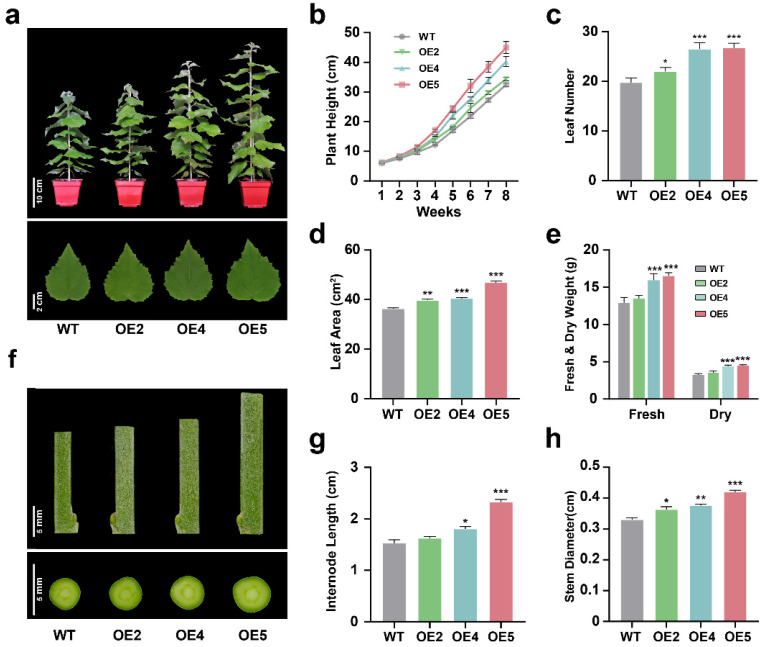
Growth comparisons of wild type (WT) and *PtGSTF1* transgenic plants. Four-week-old plants at the same size and growth state propagated on MS medium were transferred to soil and grown in a greenhouse under the same environmental conditions for 8 weeks. (**a**–**e**) Plant and leaf phenotypes, plant heights, leaf numbers, leaf areas (the first fully expanded leaves from the bottom), and shoot fresh and dry weights of WT and *PtGSTF1* overexpressing plants were examined. (**f**,**g**) Phenotypes and lengths of the 12th internodes counted from the apex. (**h**) Stem diameters. WT, wild type; OE2, OE4 and OE5, different transgenic lines. Values are the mean ± SD from five independent experiments (*n* = 5). *, *p* < 0.05; **, *p* < 0.01; ***, *p* < 0.001.

To dissect the possible role of *PtGSTF1* in the growth and development of poplar, we compared the growth phenotypes of transgenic polar plants (OE2, OE4 and OE5) with that of wild type (WT) plants. Four-week-old plants at the same size and growth state propagated on MS medium were transferred to soil and grown in a greenhouse under the same environmental condition for another 8 weeks. Significant growth phenotype difference was observed between WT and transgenic plants (lines OE2, OE4 and OE5). All transgenic plants grew faster than did the WT plants, as indicated by the drastically increased plant height, leaf number, leaf area, shoot fresh and dry weight, internode length and stem diameter (Figure 3a–h). We further assessed shoot regeneration of WT and transgenic plants. The upper parts of eight-week-old WT and transgenic plants were cut off with only a 20 cm stump left, and the plants were kept in greenhouse for one week. A faster shoot regeneration was observed on the stumps of transgenic plants (Supplementary Figure S2). These observations suggest that overexpression of *PtGSTF1* promoted the growth of transgenic poplar plants.

**Figure 4:**
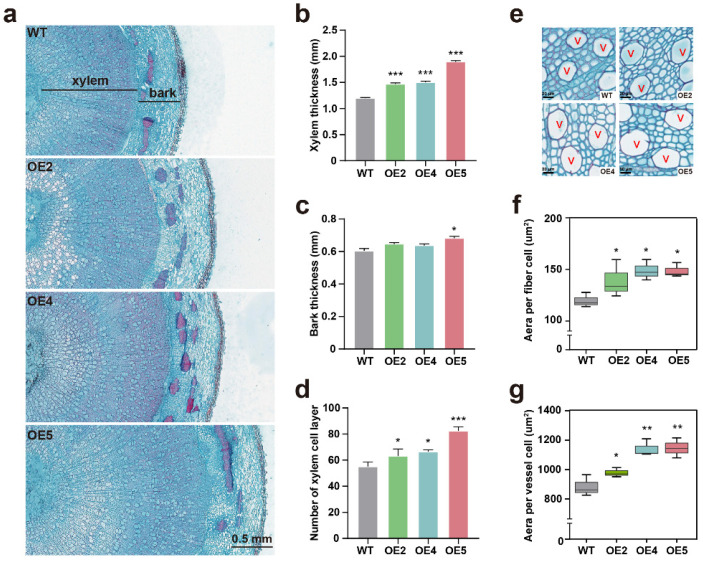
Anatomic analysis of stem growth and development of wild type and *PtGSTF1* transgenic plants. Four-week-old plants at the same size and growth state propagated on MS medium were transferred to soil and grown in a greenhouse under the same environmental conditions for 12 weeks. (**a**) Cross sections of stems showing increased xylem area in transgenic plants. (**b**,**c**) Xylem and bark thickness. (**d**) Xylem cell layer numbers. (**e**–**g**) Quantification of the size of fiber and vessel (V) cells. Images were captured on toluidine blue-stained anatomical sections. The area of fiber and vessel cells was measured and calculated via IMAGEJ based on the images. WT, wild type; OE2, OE4 and OE5, different transgenic lines. Values are means ± SD of 200 fiber or vessel cells from three plants of WT and each *PtGSTF1* transgenic line, respectively. *, *p* < 0.05; **, *p* < 0.01; ***, *p* < 0.001.

Since the stem diameters of transgenic plants were significantly increased, we compared the second growth of WT and transgenic plants. Cross sections from the basal internodes of twelve-week-old WT and transgenic plants were histochemically stained with toluidine blue for microscopic observations. Compared to WT, an increased radial width of stem and xylem was observed in the plants of all transgenic lines, as indicated by the enhanced xylem thickness (Figure 4a,b). An improved bark thickness was also seen in transgenic line OE5 plants, which showed higher *PtGSTF1* overexpression than transgenic lines OE2 and OE4 plants (Figure 2d and Figure 4c). Compared to WT plants, the increased stem growth in length and width of transgenic plants was due to both increased xylem cell number and size. The number of xylem cell layers, and the area of fiber and vessel cells in transgenic plants were remarkably greater than those in WT plants (Figure 4d–g). Quantitative measurement analyses demonstrated that the number of xylem cell layers, the cell size of xylem fibers and the cell size of vessels in transgenic plants increased 14–43%, 16–24% and 12–30%, respectively. These data suggested that overexpression of *PtGSTF1* boosted the development of xylem cell (fiber and vessel) differentiation and expansion in transgenic plants.

### 2.4. Overexpression of PtGSTF1 Confers Salt Tolerance on Transgenic Poplar

**Figure 5:**
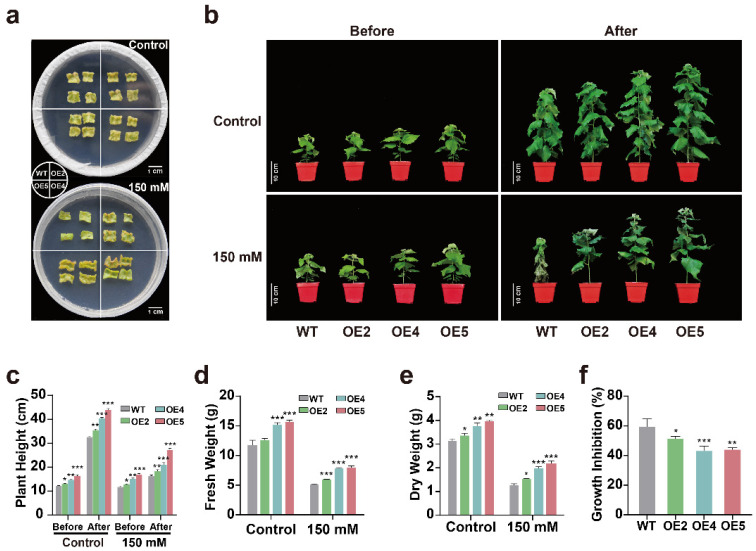
Overexpression of *PtGSTF1* enhanced salt tolerance in transgenic plants. (**a**) Callus induction analysis. Leaf explants from four-week-old wild type (WT) and *PtGSTF1* transgenic plants were cultured on callus induction medium supplemented with 0 (control) or 150 mM NaCl for 1 week. (**b**) Growth phenotypes of WT and transgenic plants. Four-week-old plants grown in a greenhouse were treated with 0 (control) or 150 mM NaCl for another 4 weeks. (**c**–**f**) Plant heights, fresh and dry weights, and growth inhibitions before and after the salt treatment. WT, wild type; OE2, OE4 and OE5, different transgenic lines. Values are the mean ± SD from three independent experiments (*n* = 3). *, *p* < 0.05; **, *p* < 0.01; ***, *p* < 0.001.

To understand how *PtGSTF1* overexpression affected the stress response of poplar plants, we examined the growth rates of WT and *PtGSTF1* transgenic plants grown under normal and salt stress conditions at both callus and whole plant scales. We first compared the callus formation on the leaf explants of WT and transgenic poplar cultured in vitro. On normal callus inducing medium (CIM), both WT and transgenic leaf explants formed callus tissues after one week. However, when they were cultured on CIM supplemented with 150 mM NaCl, callus formation on the explants of WT was apparently inhibited compared to that on the explants of transgenic plants (Figure 5a). We then compared the effects of salt stress on the whole plant growth of WT and transgenic poplars. Twenty greenhouse grown four-week-old plants of WT and each of transgenic lines (OE2, OE4 and OE5) were treated with 0 or 150 mM NaCl for another four weeks in each experimental replicate. Stably, overexpression of *PtGSTF1* led to promoted growth of transgenic poplar under normal growth conditions. Transgenic lines OE2, OE4 and OE5 all grew faster, as indicated by the significant differences in growth phenotype such as plant height and shoot biomass between WT and transgenic plants (Figure 5b–e). Transgenic lines OE2, OE4 and OE5 produced 6.42%, 22.50% and 24.84%, and 6.56%, 16.55% and 20.82% more fresh and dry shoot mass, respectively (Figure 5d,e). When treated with 150 mM NaCl, the growth of both WT and transgenic plants were considerably inhibited (Figure 5b–e). However, the growth inhibition in WT plants was dramatically more severe (Figure 5f). Transgenic plants still produced significantly greater shoot biomass than did WT plants, accompanied by a less severe salt toxic damage in the leaves (Figure 5b–e). After 4 weeks of salt treatment, almost all the leaves of WT plants, compared with only a few leaves of transgenic plants, became withered (Figure 5b). Compared to WT, transgenic lines OE2, OE4 and OE5 produced 14.09%, 25.96% and 35.82%, and 16.84%, 36.18% and 42.07% more fresh and dry shoot mass, respectively (Figure 5d,e). All these results demonstrated that *PtGSTF1* overexpression conferred salt tolerance on transgenic plants.

### 2.5. Transgenic Plants Overexpressing PtGSTF1 Accumulate Less Na^+^ and More K^+^ under Salt Stress Condition

**Figure 6:**
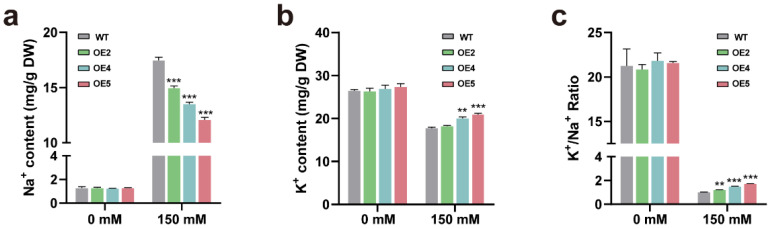
Ion content analysis. Four-week-old wild type (WT) and *PtGSTF1* transgenic plants grown in greenhouse were treated with 0 (control) or 150 mM NaCl for another 4 weeks, and the contents of Na^+^ and K^+^ were determined. (**a**) Na^+^ contents. (**b**) K^+^ contents. (**c**) K^+^/Na^+^ ratios. WT, wild type; OE2, OE4, and OE5, different transgenic lines. Values are the mean ± SD from three independent experiments (*n* = 3). ** *p* < 0.01 and *** *p* < 0.001.

The maintenance of intracellular ion homeostasis is one of the key factors that affect the normal growth of plants under salty environmental conditions. To explore whether the intracellular distribution of sodium (Na^+^) and potassium (K^+)^ play a role in the resistance of transgenic plants to salt stress, we examined the contents of K^+^ and Na^+^ in the leaves of WT and *PtGSTF1* transgenic plants after the salt treatment. In the absence of 150 mM NaCl, no significant difference in Na^+^ and K^+^ contents was observed (Figure 6a,b). In the presence of 150 mM NaCl, both WT and transgenic plants accumulated more Na^+^ and less K^+^ in the leaves. However, the level of Na^+^ and K^+^ contents in the leaves of transgenic plants was significantly lower and higher, respectively, leading to a remarkably higher K^+^/Na^+^ ratio (Figure 6a–c). This observation suggests that overexpression of *PtGSTF1* increased the salt tolerance of transgenic plants, possibly by affecting the ion homeostasis under the high salt stress condition.

### 2.6. Growth and Stress Related Gene Expression Is Up- or Down-Regulated in Transgenic Plants

**Figure 7:**
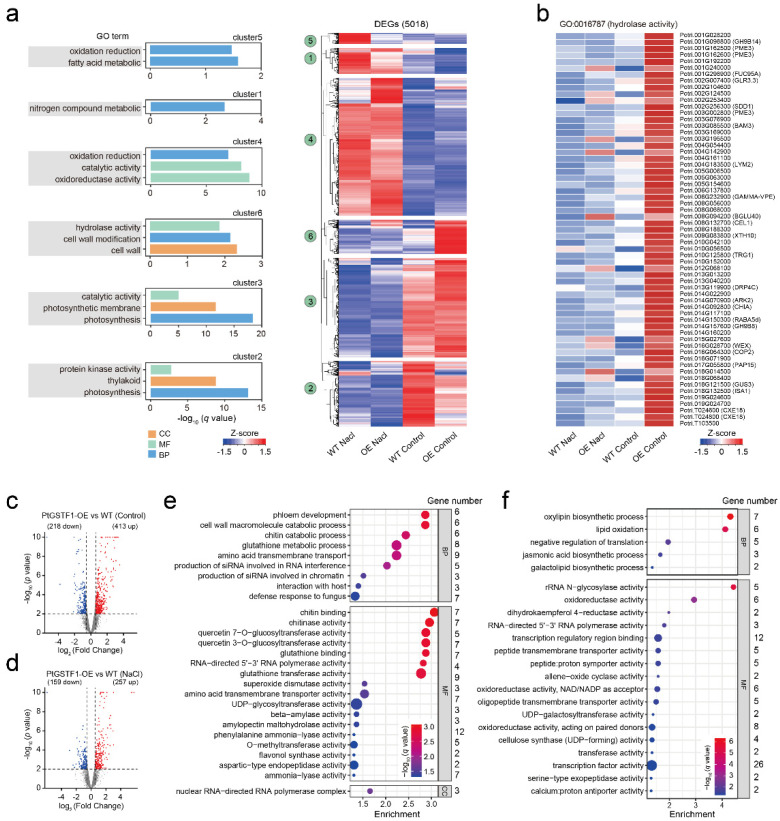
Transcriptomic profiling analysis. Four−week−old wild type (WT) and *PtGSTF1* transgenic line OE5 plants grown in a greenhouse were treated with 0 (control) or 150 mM NaCl for 48 h. The 3rd to 5th full expanded leaves from the apex were taken for RNA-seq analysis. (**a**) Hierarchical clustering and heatmap of 5018 differentially expressed genes (DEGs) in transgenic line OE5 compared to the WT plants treated with 0 and 150mM NaCl. Full results of GO enrichment analysis of heatmap DEG clusters are shown in Table S2. (**b**) Expression of genes related to hydrolase and cell wall modification in cluster 6. The Z-score scale represents mean-subtracted regularized log-transformed FPKM. (**c**,**d**) Volcano plots showing the number of DEGs under control (**c**) and salt (**d**) stress conditions. DEGs were identified using *p*-value < 0.01 and absolute fold change >1.5 as criteria. (**e**,**f**) GO annotation of DEGs between WT and transgenic line OE5 plants under control I and salt (**f**) stress conditions.

To understand how *PtGSTF1* overexpression improved the growth and salt tolerance of transgenic plants at gene expression level, we performed RNA-Seq analysis with four-week-old WT and transgenic line OE5 plants treated with 0 or 150 mM NaCl for 48 h. Differentially expressed genes (DEGs) between transgenic line OE5 and WT plants treated with 0 and 150 mM NaCl were analyzed based on the criteria fold change >1.5 and *p* < 0.01. A total of 5018 DEGs enriched into 6 gene clusters as classified with Gene Ontology (GO) term analysis were identified (Table S1). Functional annotations of individual clusters indicated that they were associated with nitrogen compound metabolic (cluster 1), photosynthesis and photosynthetic membrane (cluster 2 and 3), oxidation reduction (cluster 4 and 5), and cellulose and cell-wall metabolic processes (clusters 6) (Figure 7a). In addition, a number of genes related to hydrolase activity (GO: 0016787), which were also involved in cell wall modification, were identified in cluster 6, suggesting that overexpression of *PtGSTF1* up- or down-regulated the transcription of genes related to cell wall modification (Figure 7b).

In transgenic line OE5 plants, under the normal condition, a total number of 413 and 218 genes, whereas under the salt stress condition, a total number of 257 and 159 genes, were respectively up- or down-regulated compared to WT plants (Figure 7c,d; Tables S2 and S3). Further GO and KEGG analyses revealed that, under normal conditions, these DEGs between WT and transgenic line OE5 plants were involved in cell wall macromolecule catabolic (GO:0016998), phloem development (GO:0010088), glutathione metabolic (GO:0006749) and UDP-glycosyltransferase activity (GO:0008194), and were significantly enriched in MAPK signaling pathway, starch and sucrose metabolism, and glutathione metabolism (Figure 7e and Figure S3a). Under salt stress conditions, these DEGs between WT and transgenic line OE5 plants were involved in oxylipin biosynthetic process (GO:0031408), lipid oxidation (GO:0034440) and oxidoreductase activity (GO:0016702, GO:0016616, GO:0016709), and were significantly enriched in plant hormone signal transduction and MAPK signaling pathway (Figure 7f and Figure S3b). It is also worth noting that 35 DEGs, including 26 significantly up-regulated ones, in salt treated transgenic line OE5 plants, were enriched in the glutathione metabolism pathway compared to the untreated control (Supplementary Figure S4a,b).

To verify the accuracy and reproducibility of the RNA-seq results, we randomly selected 10 DEGs and validated their expression levels with qRT-PCR analyses. We found that, under both control and salt stress conditions, the trends of expression differences of all the 10 genes were identical to those concluded from the transcriptome data (Supplementary Figure S5a,b).

### 2.7. Overexpression of PtGSTF1 Increases ROS Scavenging Ability of Transgenic Plants

**Figure 8:**
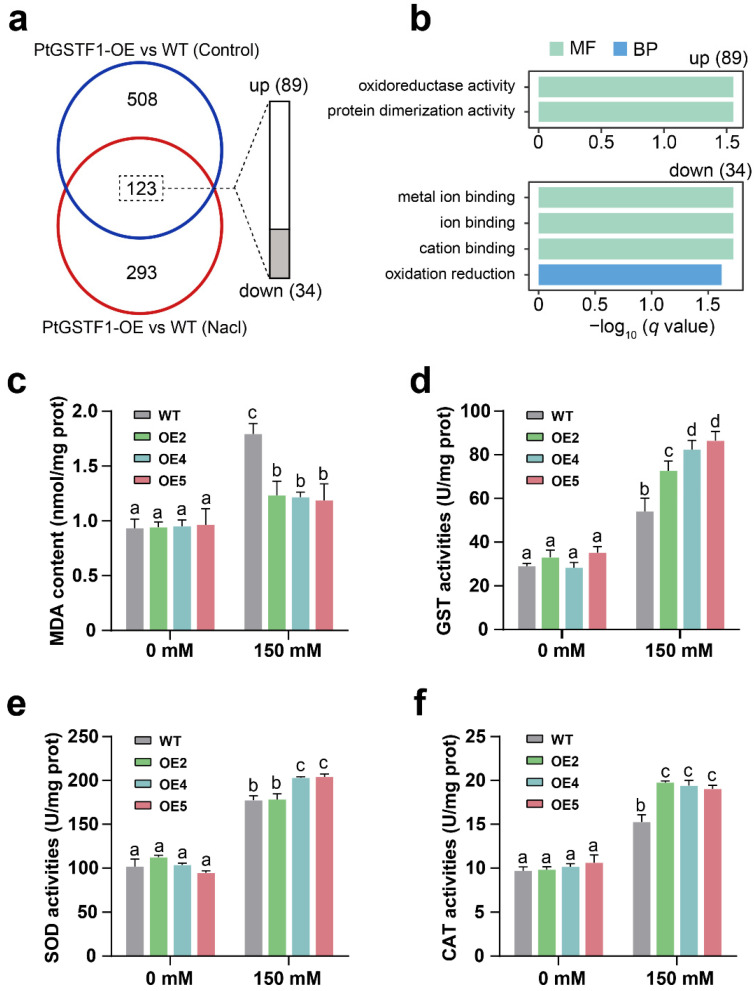
Antioxidant enzyme and glutathione S-transferase activity analyses. (**a**) Venn diagrams showing the numbers of DEGs between wild type and transgenic line OE5 plants treated with 0 and 150 mM NaCl. (**b**) GO Terms for up-regulated (89) and down-regulated (34) DEGs (FDR adjusted *p*-value < 0.05). MF, molecular function; BP, biological process. (**c**) MDA content. (**d**–**f**) GST, SOD and CAT activities. Four−week−old wild type (WT) and transgenic lines (OE2, OE4, OE5) were treated with 0 or 150 mM NaCl for 4 weeks. Values are the mean ± SD from three independent experiments (*n* = 3). The lowercase letters reflect the levels of statistical significance of one-way ANOVA with Tukey’s HSD test (*p* < 0.05).

The major function of GSTs is the detoxification of exogenously applied xenobiotics and endogenously generated organic peroxides, such as lipid peroxides [41]. We found that under the salt stress condition, the identified DEGs were significantly enriched in oxylipin biosynthetic process (GO:0031408), and lipid oxidation (GO:0034440) and oxidoreductase activity (GO:0016702, GO:0016616, GO:0016709) pathways (Figure 7f). In addition, a total number of 123 overlapped DEGs between WT and transgenic line OE5 plants, including 89 up-regulated and 34 down-regulated, were identified under both normal and salt stress conditions (Figure 8a). Interestingly, they were all significantly enriched in oxidoreductase activity (GO:0016491) (Figure 8b). To further test whether *PtGSTF1* is involved in plant tolerance to oxidative stress, we compared the content of malondialdehyde (MDA) and the activity of GST, superoxide dismutase (SOD) and catalase (CAT) in the leaves of WT and transgenic plants under both normal and salt stress conditions. Under normal growth condition, both WT and transgenic plants accumulated the same level of MDA. Upon the treatment with 150 mM NaCl, the levels of MDA content increased in both types of plants. However, the increase in WT plants was significantly higher than that in transgenic plants (Figure 8c). Similarly, both WT and transgenic plants showed the same level of GST, SOD and CAT activity under the normal growth condition, and the activity increased upon the salt treatment. Opposite to the increase of MDA content, however, the increase of GST, SOD and CAT activity in WT plants was significantly lower than that in transgenic plants (Figure 8d–f). These results indicated that overexpression of *PtGSTF1* enhanced the tolerance of transgenic poplar to salt induced oxidative stress.

### 2.8. Genes Related to ABA Biosynthesis and Signal Transduction Are Up-Regulated in Transgenic Plants under Salt Stress Conditions

**Figure 9:**
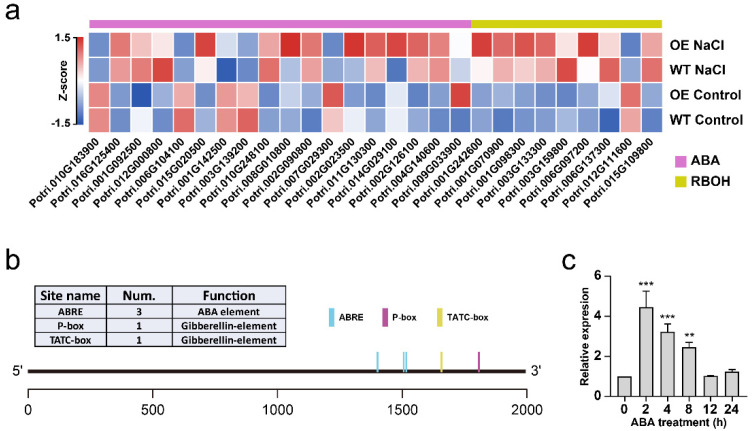
*PtGSTF1* expression was responsive to ABA treatments. (**a**) Expression of ABA (purple callout) and RBOH−related (yellow callout) DEGs. (**b**) *PtGSTF1* promoter structure analysis with a transcription start site at the +1 position and potential cis-acting regulatory DNA elements (according to the PLACE database). The 2 kb upstream sequence of *PtGSTF1* was selected. (**c**) Expression of *PtGSTF1* upon ABA treatment. Four-week-old wild type Shanxin yang plants were treated with 0 or 100 mM ABA for 0, 2, 4, 8, 12 and 24 h. Values are the mean ± SD from three independent experiments (*n* = 3). ** *p* < 0.01 and *** *p* < 0.001.

In the regulation of plant growth and stress tolerance, the action of plant hormones is closely integrated with the production and signaling of ROS. Under stress condition, ABA can activate respiratory burst oxidase homolog (RBOH) to induce the production of ROS. We noticed that in the DEGs between WT and transgenic plants under both normal and salt stress conditions, a number of ABA and RBOH related genes were also identified. The expression of genes related to ABA biosynthesis (*Potri.001G092500*, *Potri.001G142500*, *Potri.002G023500*, *Potri.002G090800*, *Potri.002G126100*, *Potri.003G139200*, *Potri.004G140600*, *Potri.006G104100*, *Potri.007G029300*, *Potri.008G010800*, *Potri.009G033900*, *Potri.010G183900*, *Potri.010G248100*, *Potri.011G130300*, *Potri.012G000800*, *Potri.014G029100*, *Potri.015G020500* and *Potri.016G125400*) and signal transduction (*Potri.001G242600*, *Potri.001G070900*, *Potri.001G098300*, *Potri.003G133300*, *Potri.003G159800*, *Potri.006G097200*, *Potri.006G137300*, *Potri.012G111600* and *Potri.015G109800*), as well as genes encoding RBOHs, was remarkably up-regulated in transgenic plants, especially under the salt stress condition (Figure 9a). Consistently, multiple plant hormone responsive cis-elements, including 3 ABREs (ABA response), and 1 P-box and 1 TATC-box (gibberellin response) elements, were found in the promoter region of *PtGSTF1* (Figure 9b). Therefore, the expression of *PtGSTF1* might be responsive to ABA. To verify this conjecture, we performed qRT-PCR analysis. We found that similar to the expression pattern with the treatment of 150 mM NaCl, the *PtGSTF1* expression was also significantly induced by ABA in the leaves of four-week-old wild type Shanxin yang plants treated with 100 mM ABA. Within 2 h of the treatment, a three fold increase of *PtGSTF1* expression was observed (Figure 9c). All these observations imply that the expression of *PtGSTF1* was responsive to both ABA and salt stress.

## 3. Discussion

In plants, multiple members of GSTs have been identified and their roles in shoot morphogenesis have been respectively demonstrated in vivo and in vitro [42,43]. To understand their biological functions in the growth and stress response in tree crops, we isolated *PtGSTF1* gene from poplar, which shared high similarity with other GST members from different plant species (Figure 1a and Figure S1). *PtGSTF1* was abundantly expressed in leaves and was responsive to high salt stress (Figure 1b,c). This is similar to the expression patterns of other previously reported GST members, which showed induced expression by various biotic and abiotic stresses in different biological metabolism processes [16,31,33,34,44,45]. In Arabidopsis, GSTU7 was reported to be localized in the cytosol, whereas the tea CsGSTU8 was found to be distributed throughout the cell [16,17,46,47]. We observed that PtGSTF1 was located in the cytoplasm, implying that it may have a similar role to the GSTU7 protein (Figure 1c).

The functions of GSTs in plant growth and development have been reported in several studies. Heterologous expression of the rice *OsGSTL2* in Arabidopsis led to promoted flowering and improved abiotic stress tolerance of transgenic plants [34]. The loss of function mutation of *GSTU7* in the Arabidopsis *gstu7* null mutants led to restrained growth and delayed onset of the MV-induced antioxidative response [16]. To investigate the possible role of GSTs in the growth and resistance to abiotic stress in tree plants, we generated transgenic poplar with different *PtGSTF1* overexpression levels (Figure 2a–d). Overexpression of *PtGSTF1* drastically accelerated the growth of transgenic plants, leading to increased leaf and stem biomass compared to the WT plants (Figure 3a–h). Coincidently, similar growth change was also shown in the reported *gstu7/GSTU7-V5* complemented lines [16]. Compared to WT, the plant sizes of complementary lines #2 and #5 were significantly bigger. We speculate that the increased growth of these complementary lines could be due to the overexpression of *GSTU7* driven by the strong *UBQ10* promoter in the *gstu7* mutant.

To clarify how *PtGSTF1* overexpression affected the stem growth of transgenic plants, we performed anatomic examinations and transcriptomic analyses. Compared to WT, the proportion of woody tissues of transgenic plants overexpressing *PtGSTF1* was significantly greater. More detailed analyses revealed that *PtGSTF1* overexpression enhanced both the number and size of xylem cells (Figure 4a–g). This is different from observations in transgenic poplar overexpressing *CYP85A3*, in which the promoted stem growth resulted from the increased xylem cell number only, implying that the secondary xylem development in *PtGSTF1* transgenic plants was promoted differently [48].

In Arabidopsis plants ectopically expressing *OsGSTL2*, the resistance of transgenic seedlings to heavy metal, osmotic, cold and salt stress was dramatically improved compared to WT [34]. We also observed that transgenic poplar plants not only showed promoted shoot growth, but also showed improved resistance to salt stress, accompanied with a lower Na^+^ and higher K^+^ accumulation in the leaves under high salt stress conditions (Figure 5a–f and Figure 6a–c). Similar results were also observed in transgenic poplar plants overexpressing the H^+^-pyrophosphatase gene *PtVP1.1* and the constitutively active form of *PtSOS2* gene *PtSOS2TD* [49,50]. The net uptake of Na^+^ depends on its influx, exclusion and sequestration, as well as other Na^+^ regulation processes, such as the loading and unloading of Na^+^ in xylem and the recycling of Na^+^ in phloem. Excessive ions in plant cells interfere with normal cellular metabolism and have toxic effects on normal growth and development. Therefore, under salinity stress conditions, maintenance of intracellular K^+^ and Na+ homeostasis is very important for the normal growth of plants [51,52,53]. Plant cells can accumulate K^+^ and excrete Na^+^ to maintain a high K^+^/Na^+^ ratio, which helps to mitigate the toxic effects of salt, thereby improving salt tolerance [54,55,56]. The results reported in this study indicated that overexpression of *PtGSTF1* effectively restricted the entry of Na^+^ and protected enzymatic processes in the cytoplasm by decreasing the level of Na^+^ under salt stress conditions.

Excess cytoplasmic Na^+^ can also lead to the production of ROS such as singlet oxygen, H_2_O_2_ and O^2−^, which causes membrane lipid peroxidation [47,50,57]. We found that, under both normal and salt stress conditions, a number of genes related to nitrogen compound metabolism, photosynthesis and photosynthetic membrane, oxidation reduction, and cellulose and cell-wall metabolic process were up- or down-regulated in *PtGSTF1* overexpressing plants (Figure 7a–f and Figure 8a,b). The content of MDA and the activity of GST and antioxidant enzymes were examined in WT and transgenic plants overexpressing *PtGSTF1*. The accumulation of MDA in the leaves of transgenic plants was significantly lower than that in the leaves of WT plants upon the exposure to salinity stress (Figure 8c). The mechanisms of enzymatic (catalase, peroxidase, superoxide dismutase) and non-enzymatic (glutathione, GSH; ascorbate, ASA) response have been identified as abiotic stress for NaCl detoxification. As the central signaling molecule, ROS plays an important role in the process of plant defense responses and salt stress tolerance [26,58,59]. Through the development and evolutionary expansion of a series of enzymatic and non-enzymatic ROS scavengers, cell damage caused by ROS has been effectively alleviated [60]. ROS scavenging enzymes, such as GST, CAT, SOD, APX, and enzymes related to the glutathione cycle, have important functions in maintaining normal cellular ROS homeostasis. GSTs catalyze the conjugation of GSH to a range of hydrophobic and electrophilic substrates including ROS in the ROS scavenging system, and thus protect the cells from oxidative burst [61,62]. They also catalyze the interaction between GSH and hydrogen peroxide, thereby regulating the perception and tolerance of various abiotic stressors in plants [63]. During catalysis, the binding and proper orientation of GSH are controlled by the conserved GSH binding site (G-site), and the substrate binding pocket (H-site) assists in the binding of substrates by providing a hydrophobic environment [61]. When subjected to salt stress, *PtGSTF1* overexpressing plants exhibited a stress tolerant phenotype, accompanied with a higher antioxidant enzyme activity, indicating the positive role of *PtGSTF1* in ROS scavenging (Figure 8d–f). *PtGSTF1* harbors both the conserved GSH binding site and substrate binding pocket, suggesting the capacity of *PtGSTF1* to catalyze the conjugation of GSH to an array of substrates [8]. Therefore, the higher oxidative stress resistance in transgenic plants under salt stress condition can be explained by their increased GSH-ROS binding capacity and ROS scavenging ability.

ABA plays a crucial role in plant resistance to abiotic stress in conjunction with glutathione transferase [30,64,65]. Unlike other GSTFs such as *AtGSTF2* in particular, *PtGSTF1* was unable to interact or activate with auxin and other heterocyclic compounds such as noramine, indole-3-aldehyde and quercetin, although no dramatic structural difference was observed between *PtGSTF1* and *AtGSTF2* [8,66,67]. However, this does not mean that *PtGSTF1* could not interact or activate with other hormones. Indeed, our transcriptomic data showed that a number of ABA biosynthesis and signal transduction related genes were up-regulated in transgenic plants under salt stress conditions (Figure 9a). Coincidently, several ABA responsive cis-elements, including three ABREs (ABA response), were found in the promoter region of *PtGSTF1*, implying that *PtGSTF1* expression might be induced in response to ABA (Figure 9b). Our qRT-PCR assays confirmed the inducible expression of *PtGSTF1* by ABA (Figure 9c). All these results further imply that the expression of *PtGSTF1* was induced in response to salt stress and ABA treatment.

Taken together, we characterized the role of *PtGSTF1* in poplar growth and salt stress tolerance in this work. Overexpression of *PtGSTF1* in poplar resulted in enhanced biomass production and salt tolerance, as indicated by the increased xylem cell number and size, optimized Na^+^/K^+^ homeostasis, and improved scavenging of reactive oxygen species (ROS) in transgenic plants grown under a salt stress condition. Further transcriptome analysis showed that overexpression of *PtGSTF1* altered the expression of hydrolase, cell wall modification, and ion and ROS homeostasis related genes under normal or a salt stress condition. Our findings indicate that *PtGSTF1* has a great potential to be used as a candidate gene for the future breeding of new tree species with increased growth and salt resistance.

## 4. Materials and Methods

### 4.1. Plant Materials, Growth Condition and Transformation

The hybrid clone Shanxin yang (*Populus davidiana* × *Populus bolleana*) was used. The poplar materials were aseptically cultured on MS medium supplemented with 0.1 mg/L naphthalene acetic acid (NAA). Gene-specific primers 5′-ATGGCAACTCCGGTGAC-3′ and 5′-TCAAGCATTTT TCCTCATTTC-3′ were used to isolate the coding sequence of *PtGSTF1* (Potri.002G015100) by PCR amplification. *PtGSTF1* coding sequence was subsequently inserted into a modified pCAMBIA2301 vector via the *Sal* I and *Bam* H I restriction sites for plant expression under the control of cauliflower mosaic virus (CaMV) 35S promoter. Primer sequences used for the construction of plant transformation vector are listed in Table S4. The resultant construct was introduced into *Agrobacterium tumefaciens* (*A. tumefaciens*) strain EHA105 for poplar transformation as described previously [68]. Regenerated transgenic poplar plants were propagated and transplanted into soil as described in our previous report [69]. Plantlets were grown in the culture room with cool white fluorescent light (~200 μmol/m^−2^/s^−1^) under a 12-h light/dark photoperiod at 21–25 °C/15–18 °C (day/night). For plants grown in a greenhouse, four-week-old plantlets were transferred to soil and grown under a 14-h light/dark photoperiod comprising natural daylight supplemented with lamps (120–150 μmol/m^−2^/s^−1^) at about 21–25 °C/15–18 °C (day/night).

### 4.2. Histochemical Staining Analyses

To confirm the expression of the co-transformed reporter gene (GUS) with *PtGSTF1*, histochemical staining was conducted as described previously [70]. Leaves of three-week-old wild type (WT) and transgenic poplar plants were soaked in GUS staining solution (0.5 M Tris, pH 7.0, 10% Triton X-100 with 1 mM X-Gluc (5-bromo-4-chloro-3-indolyl-D-glucuronide)) for 15 min at 37 °C in dark. After the reaction, photosynthetic pigments were removed with 75% ethanol at room temperature.

### 4.3. Salt Tolerance Analysis

For salt stress analyses, four-week-old micro-propagated wild type (WT) and *PtGSTF1*-overexpressing (*PtGSTF1*-OE) plants were transplanted into soil and grown in a greenhouse. All the plants were irrigated weekly with liquid 1/8 MS. After four weeks, 20 healthy plants at the same growth size and state of WT and each transgenic line were watered with 1/8 MS supplemented with 0 (control) or 150 mM NaCl. After another four weeks, plant material samples were collected for physiological index measurement.

### 4.4. Subcellular Localization of PtGSTF1 Protein

To assay the subcellular localization of PtGSTF1 protein, the 648 bp coding sequence was amplified and inserted into pBIN-GFP vector at the C-terminal of GFP and introduced into *A. tumefaciens* GV3101. Bacterial cells harboring 35S::GFP-*PtGSTF1* and 35S::GFP were cultured, centrifuged and adjusted to an OD600 of 0.8 in re-suspending buffer (10 mM MES, 10 mM MgCl_2_ and 100 mM acetosyringone; pH 5.7). After 2 h incubation at 25 °C, the cultures were transiently expressed in four-week-old tobacco (*Nicotiana benthamiana*) leaves, and GFP signal was observed with a LECIA Automatic Fluorescence Microscope (LECIA, Wetzlar, Germany) 48 h after the infiltration.

### 4.5. ABA Treatment Analysis

Four-week-old wild type Shanxin yang plants at the same growth size and state were respectively treated with 0 or 100 mM ABA for 0, 2, 4, 8, 12 and 24 h. A total amount of 0.5 mL ABA was sprayed on the leaves of each plant. The first to third leaves from each plant were collected for RNA extraction after different treatments. Three biological replicates were performed for all the treatments.

### 4.6. Na^+^ and K^+^ Content Determination

The ion contents in the leaves of poplar plants were assessed. Four-week-old wild type and *PtGSTF1*-OE plants grown in a greenhouse were treated with 0 mM or 150 mM NaCl for 4 weeks and mature leaves were collected to determine the contents of Na^+^ and K^+^ with some modifications as described previously [71]. Each dry leaf sample (0.5 g) was incubated with 1 mL nitric acid, extracted in a boiling water bath for 3 h, filtered and diluted with 10 mL ultrapure water. Ion contents were measured using an AP1500 flame photometer (Shanghai Aopu Analytical Instruments Co., Ltd., Shanghai, China).

### 4.7. Cross-Sectioning and Histological Staining of Poplar Plants

For histological observations, stems from the basal parts of twelve-week-old WT and transgenic plants grown in a greenhouse were fixed with FAA solution and embedded in paraffin. Four micrometer thick sections were cut out with a rotary microtome (Leica RM2235, Wetzlar, Germany) and stained with 0.05% (*w*/*v*) toluidine blue for 5 min. Cross sections were observed using a microscope. Images were captured under bright field using a Nikon Ci-S microscope (Nikon, Tokyo, Japan). The radial widths of phloem and xylem as well as morphological parameters of xylem cells were measured using the IMAGEJ.

### 4.8. RNA-Seq Assays

For RNA-seq analysis, four-week-old greenhouse grown WT and transgenic plants (line OE5) at the same growth size and state were treated with 0 or 150 mM NaCl for 48 h. Then the third mature leaf from at least five plants of each WT and transgenic line, respectively, were collected and pooled together for each biological replicate. Collected plant materials were immediately frozen in liquid nitrogen, and stored at −80 °C. Total RNA was isolated using Plant RNA Kit (Omega, Guangzhou, China) and RNA integrity was evaluated using a Bioanalyzer 2100 (Agilent). RNA concentration and purity were measured using NanoDrop 2000 (Thermo Fisher Scientific, Wilmington, DE, USA). RNA integrity was assessed using the RNA Nano 6000 Assay Kit of the Agilent Bioanalyzer 2100 system (Agilent Technologies, CA, Santa Clara, USA). Sequencing libraries were generated using NEB Next UltraTM RNA Library Prep Kit for Illumina (NEB, San Diego, USA) following the manufacturer’s instruction. Afterwards, the libraries were sequenced on an Illumina HiSeq 2500 platform. Raw data (raw reads) of fastq format were first subjected to quality control using FastQC (v.0.11.9). Then reads were mapped to the *Populus trichocarpa* (v3.0: https://phytozome.jgi.doe.gov/pz/portal.html, accessed on 10 January 2022) using HISAT2 (v.2.0.4) with default parameters. FPKM of each gene was calculated using StringTie (v.2.2.1). The analysis of differentially expressed genes (DEGs) was performed using DESeq2 (v.1.6.3) with default parameters. Significant DEGs were identified as those with a *P* value (one-way ANOVA test) of differential expression above the threshold (|Fold Change| > 1.5, *p* < 0.01). GO enrichment was performed with the accession numbers of significant DEGs via agriGO v2.0 (http://systemsbiology.cau.edu.cn/agriGOv2, accessed on 27 January 2022).

### 4.9. Malondialdehyde Content, Glutathione S-Transferase and Antioxidant Enzyme Activity Assays

Four-week-old wild type and transgenic plants were treated with 0 mM or 150 mM NaCl for 4 weeks, the first fully expanded leaves were collected for malondialdehyde (MDA) content, glutathione *S*-transferase and antioxidant enzyme activity assays. MDA content was determined with the thiobarbituric acid (TBA) chromatometry method, as described previously [72]. Leaf sample (0.5 g) was homogenated with 5 mL of 10% trichloroacetic acid (TCA) solution. After centrifugation at 6000 rpm for 10 min, 2 mL supernatant was transferred to a tube and mixed with 2 mL 0.6% TBA solution, incubated in boiling water for 15 min, then quickly cooled to room temperature. The absorbance at 532 nm was measured. The activities of GST (glutathione *S*-transferase), SOD (superoxide dismutase) and CAT (catalase) were determined using commercial kits following the manufacturer’s instruction (Nanjing Jiancheng Bioengineering Institute, Nanjing, China). GST activity was measured at 412 nm. SOD activity was determined using the xanthine/xanthine oxidase method based on the production of O^2−^ anions. CAT activity was measured by analyzing the rate at which it caused the disintegration of H_2_O_2_ at 405 nm. The generation of nicotinamide adenine dinucleotide phosphate was measured spectrophotometrically at 290 nm.

### 4.10. RNA Isolation, PCR and qRT-PCR Analysis

For *PtGSTF1* expression pattern analysis under normal growth condition, various tissues and organs from eight-week-old Shanxin yang plants grown in a greenhouse were collected. For gene expressions related to cellulose and lignin synthesis, stem samples were tested. For salt related genes under normal and salt conditions, leaf samples were used. Total RNA was extracted using Plant RNA Kit (Omega, Guangzhou, China). An amount of 1 µg total RNA was used to synthesize single-strand complementary DNA (cDNA) using HiScript II Q RT SuperMix with gDNA Eraser (Vazyme Biotech, Nanjing, China). Quantitative real time-PCR (qRT-PCR) reactions were performed using 100 ng cDNA as template with gene-specific primers and the SYBR Premix Ex Taq (Takara, Dalian, China) on BIO-RAD CFX Connect Real-Time System (Bio-Rad, Hercules, CA, USA). All the primer sequences used in this study are listed in Table S4.

### 4.11. Statistical Analyses

For statistical analyses, normality of the data was tested using the shapiro function. One-way ANOVA with Tukey’s test was performed using multcomp (v 1.4-16, Torsten Hothorn, Zurich, Switzerland) package in R. All values are represented as means ± standard deviations.

## 5. Patents

This section is not mandatory but may be added if there are patents resulting from the work reported in this manuscript.

## Data Availability

The sequencing data generated in this study has been deposited in NCBI BioProjects database with the accession number PRJNA810956 (https://www.ncbi.nlm.nih.gov/bioproject/810956, accessed on 28 February 2022).

## Supplementary Materials

The following supporting information can be downloaded at: https://www.mdpi.com/article/10.3390/ijms231911288/s1.
