# Supplementary figures and images for: The Glutathione S-Transferase PtGSTF1 Improves Biomass Production and Salt Tolerance through Regulating Xylem Cell Proliferation, Ion Homeostasis and Reactive Oxygen Species Scavenging in Poplar
Source: Int J Mol Sci. 2022 Sep 25;23(19):11288. doi: 10.3390/ijms231911288 (PMC9569880; doi:10.3390/ijms231911288)

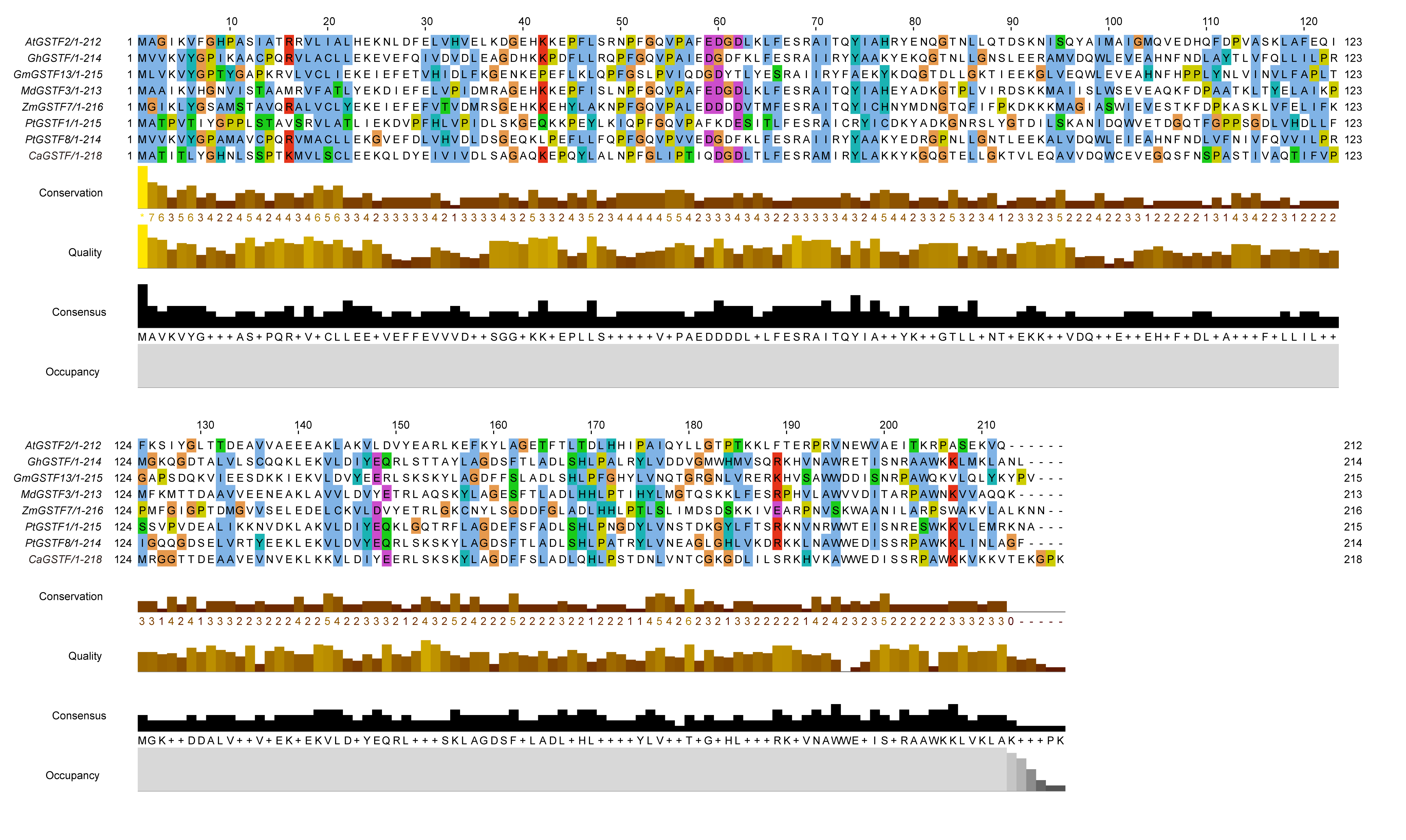

Supplement: Supplementary file 1 [file ijms-23-11288-s001.zip › Figure S1.tif]

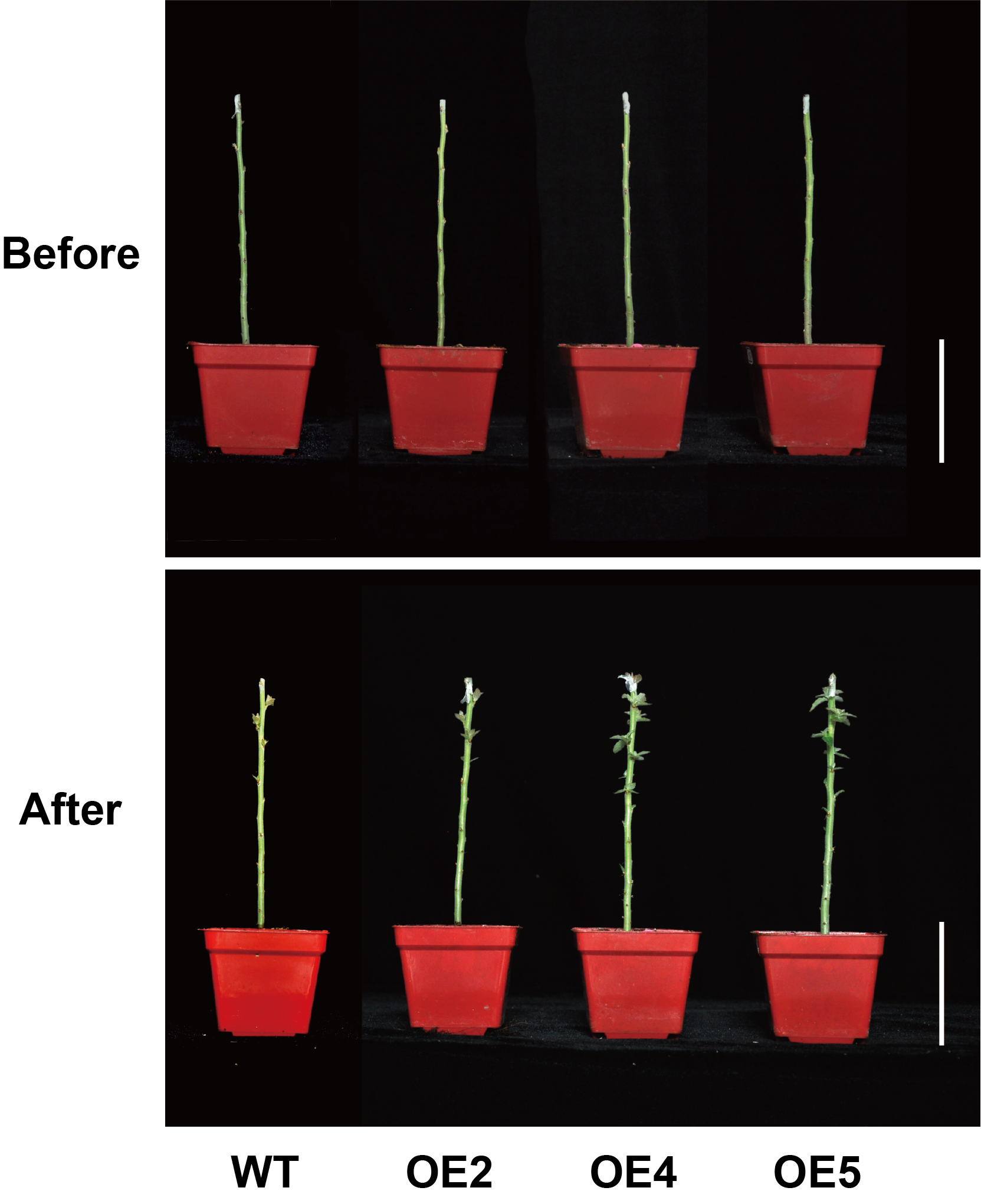

Supplement: Supplementary file 1 [file ijms-23-11288-s001.zip › Figure S2.tif]

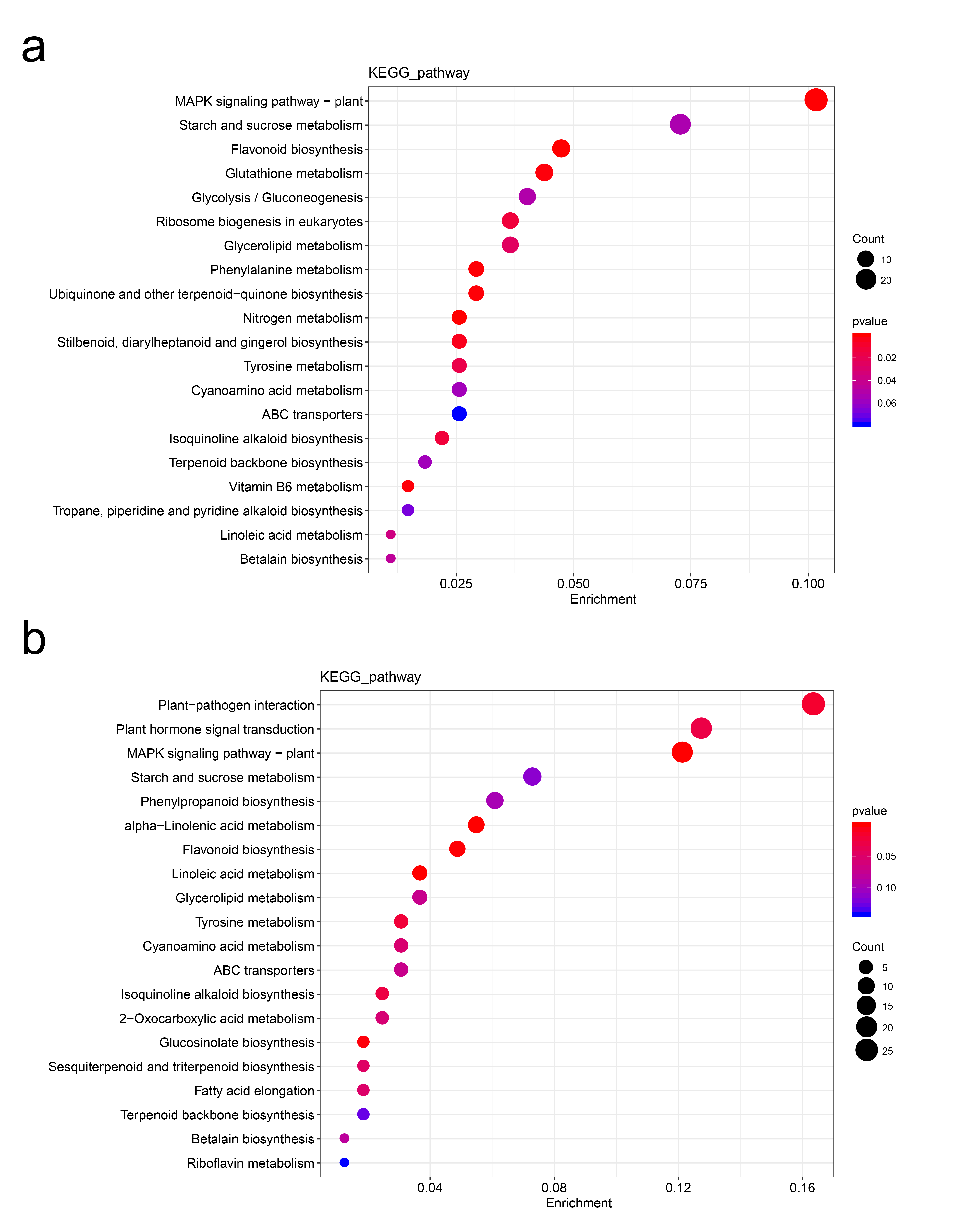

Supplement: Supplementary file 1 [file ijms-23-11288-s001.zip › Figure S3.tif]

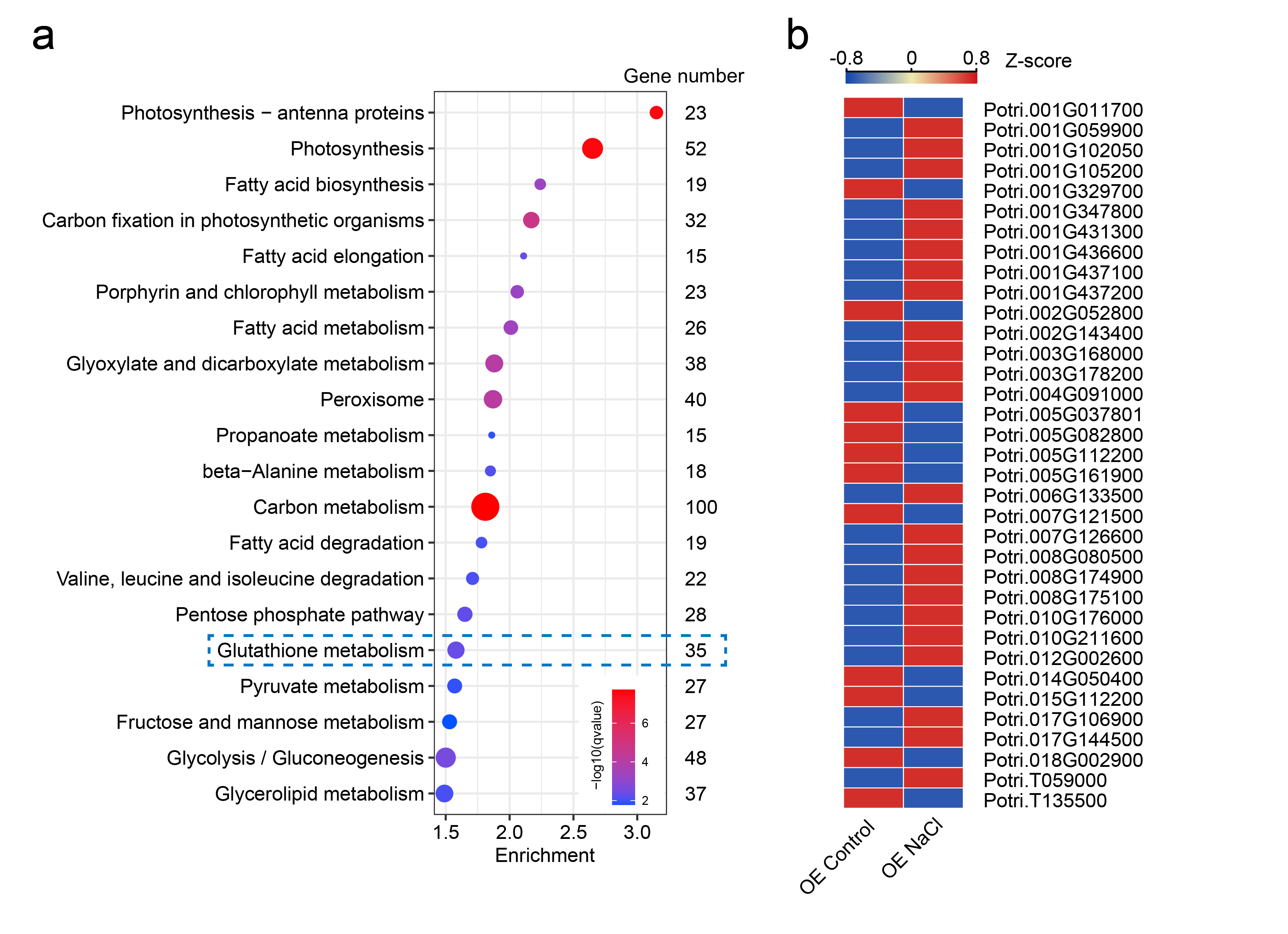

Supplement: Supplementary file 1 [file ijms-23-11288-s001.zip › Figure S4.tif]

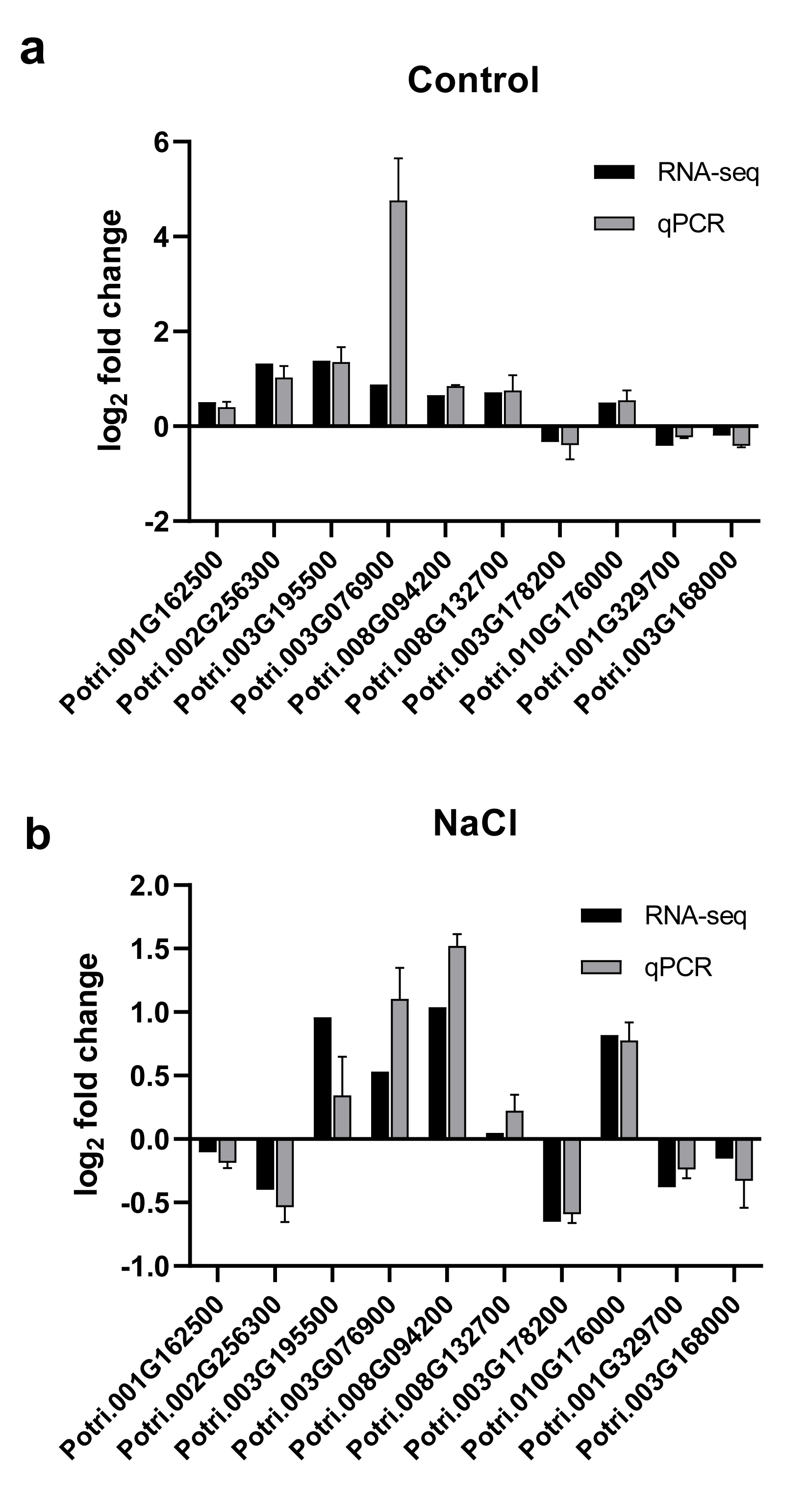

Supplement: Supplementary file 1 [file ijms-23-11288-s001.zip › Figure S5.tif]
